# Time-dependent event accumulation in a cardiovascular outcome trial of patients with type 2 diabetes and established atherosclerotic cardiovascular disease

**DOI:** 10.1186/s12933-023-01802-x

**Published:** 2023-03-28

**Authors:** M. Angelyn Bethel, Harald Sourij, Susanna R. Stevens, Karen Hannan, Yuliya Lokhnygina, Amanda I. Adler, Eric D. Peterson, Rury R. Holman, Renato D. Lopes

**Affiliations:** 1 Diabetes Trials Unit, Radcliffe Department of Medicine, University of Oxford, Oxford, UK; 2 Interdisciplinary Metabolic Medicine Trials Unit, Division of Endocrinology and Diabetology, Medical University of Graz, Graz, Austria; 3 Duke Clinical Research Institute, Duke University School of Medicine, Durham, NC USA; 4 Institute of Metabolic Science, Addenbrooke’s Hospital, Cambridge, UK; 5 Present Address: Eli Lilly & Co., Indianapolis, IN USA; 6 Present Address: Division of Cardiology, UT Southwestern Medical Center, Dallas, TX USA

**Keywords:** Type 2 diabetes, Cardiovascular outcomes, Adjudication, Myocardial infarction, Stroke

## Abstract

**Background:**

Estimating cardiovascular (CV) event accrual is important for outcome trial planning. Limited data exist describing event accrual patterns in patients with type 2 diabetes (T2D). We compared apparent CV event accrual patterns with true event rates in the Trial Evaluating Cardiovascular Outcomes with Sitagliptin (TECOS).

**Methods:**

Centrally adjudicated event dates and accrual rates for a 4-point major adverse CV event composite (MACE-4; includes CV death, nonfatal myocardial infarction, nonfatal stroke, or unstable angina hospitalization), MACE-4 components, all-cause mortality (ACM), and heart failure hospitalization were compiled. We used three graphical methods (Weibull probability plot, plot of negative log of the Kaplan–Meier survival distribution estimate, and the Epanechnikov kernel-smoothed estimate of the hazard rate) to examine hazard rate morphology over time for the 7 outcomes.

**Results:**

Plots for all outcomes showed real-time constant event hazard rates for the duration of the follow-up, confirmed by Weibull shape parameters. The Weibull shape parameters for ACM (1.14, 95% CI 1.08–1.21) and CV death (1.08, 95% CI 1.01–1.16) were not sufficiently > 1 as to require non-constant hazard rate models to accurately depict the data. The time lag between event occurrence and event adjudication being completed, the adjudication gap, improved over the course of the trial.

**Conclusions:**

In TECOS, the nonfatal event hazard rates were constant over time. Small increases over time in the hazard rate for fatal events would not require complex modelling to predict event accrual, providing confidence in traditional modelling methods for predicting CV outcome trial event rates in this population. The adjudication gap provides a useful metric to monitor within-trial event accrual patterns.

***Clinical trial registration*:**

Clinicaltrials.gov NCT00790205.

**Supplementary Information:**

The online version contains supplementary material available at 10.1186/s12933-023-01802-x.

## Introduction

Large randomized cardiovascular outcome trials (CVOTs) in type 2 diabetes (T2D) have changed the landscape of diabetes care, providing more complete evidence about the risk–benefit profile of glucose-lowering medications. Critical to trial design, planning, and conduct is having a well-considered power calculation and sample size estimate, which inform trial size and duration. In an event-driven trial, understanding true event accrual rates and the efficiency of the trial processes to collect and verify events is also critical during design and participant follow-up to appropriately set the budget, distribute trial site and monitoring resources, monitor predicted versus actual event rates, and plan end-of-trial activities.

Typically, data from previously completed trials conducted in similar populations are used to model predicted event rates, using assumptions of static event rates over time. However, scarce objective evidence exists to support those modelling assumptions. An alternate hypothesis might suggest increasing event rates over time if clinical trial participants are assumed to be healthier than the population they represent as a result of the inclusion criteria or self-selection for trial participation. Such selection could produce initial within-trial event rates lower than anticipated but increasing as the trial progresses, driven by aging and accumulation of comorbidities. Alternatively, in a time-to-first-event paradigm, apparent event rates could decrease over time if a competing risk for death exists. Understanding event accrual rates during trial follow-up is further complicated by continuous recruitment, which produces non-linear accumulation of patient-years and events, and by fluctuation in the efficiency of the processes by which events are identified and confirmed, usually through adjudication.

Here, we examine both the true event rates and the within-trial event accrual pattern over time for confirmed first events of 4-point major adverse cardiovascular (CV) events (MACE-4; defined as CV death, nonfatal myocardial infarction, nonfatal stroke, or hospitalization for unstable angina), and key secondary endpoints including the individual MACE-4 components, hospitalization for heart failure (hHF), and all-cause mortality (ACM) in the Trial Evaluating Cardiovascular Outcomes with Sitagliptin (TECOS).

## Methods

Because of the sensitive nature of the data collected for this study, requests to access the dataset from qualified researchers trained in human subject confidentiality protocols may be submitted at dcri.org/data-sharing.

### Study design

The design, protocol, and primary results of TECOS (NCT00790205) have been published previously [1, 2]. The study was designed and run independently by the Duke Clinical Research Institute (DCRI) and the University of Oxford Diabetes Trials Unit (DTU) in an academic collaboration with the sponsor (Merck Sharp and Dohme). The protocol was approved by the ethics committees associated with all participating trial sites, with all participants providing written informed consent. Briefly, 14,671 participants from 38 countries were enrolled between December 2008 and July 2012. Eligible participants were ≥ 50 years old with T2D, atherosclerotic CV disease, and HbA_1c_ values of 6.5–8.0% (48–64 mmol/mol) on stable dose mono- or dual-combination therapy with metformin, pioglitazone, sulfonylurea, or insulin (with or without metformin). Study participants were randomized in a double-blind fashion to sitagliptin or placebo at doses appropriate for their estimated glomerular filtration rate (eGFR). Patients with an eGFR < 30 mL/min per 1.73 m^2^ were not eligible. Trial visits occurred at months 4, 8, and 12, and thereafter every 6 months. Data regarding death, hospitalizations, and CV events were recorded at all visits. All reported events of death, myocardial infarction, stroke, hospitalization for unstable angina, hHF, acute pancreatitis, and cancer (other than nonmelanoma skin cancers) were adjudicated by an independent committee blinded to randomized treatment assignment. Adjudicated event definitions have been published previously [1].

### Event reporting and adjudication

Trial events were reported by investigators using an electronic case report form that captured the date events occurred, the date they were reported, and relevant clinical details. Events were adjudicated by an independent committee, blinded to treatment allocation. Upon receipt of a site-reported event, an adjudication packet was assembled, containing prespecified clinical information according to event type. Completed packets were distributed to trained adjudicators, and following review, an adjudication decision was recorded in the trial database.

### Event accrual patterns

For this analysis, the following time windows were calculated: time from event occurrence to site reporting, time from site report to adjudication packet complete (marked by packet sent for review), and time for adjudication to be complete (marked by adjudication decision recorded). Total adjudication time was the time from event occurrence to a recorded adjudication decision. Time windows are reported as median (25th, 75th percentile) and were calculated by event type and according to the phase of trial operation: recruitment (December 16, 2008–July 31, 2012), follow-up (August 1, 2012–May 4, 2014), or close-out (May 5, 2014–March 30, 2015).

### True event rate analysis

Adjudicated event data were compiled for the primary MACE-4 composite and key secondary endpoints (fatal or nonfatal myocardial infarction, fatal or nonfatal stroke, CV mortality, ACM, hospitalization for unstable angina, and hHF) from the intention-to-treat population. To determine whether the hazard rate for each endpoint was constant over time, parametric models utilizing actual event dates were constructed for each endpoint using PROC LIFEREG with DISTRIBUTION = WEIBULL without any independent variables; as part of the model output, Weibull shape parameter was estimated with 95% confidence intervals (CIs). If the 95% CI contained 1, the Weibull model was reduced to an exponential, or constant hazard rate, model. Three further graphical methods were used to evaluate the exponential model fit: (1) plots of the negative log of Kaplan–Meier survival distribution estimate (− log[S(t)]) vs. time, (2) probability plots from fitting the Weibull model, in order to evaluate the Weibull model fit and therefore confirm that Weibull shape parameter can be used to describe the data distribution, and (3) Epanechnikov kernel-smoothed estimate of the hazard rate plotted against time.

## Results

### Event accrual patterns

**Fig. 1:**
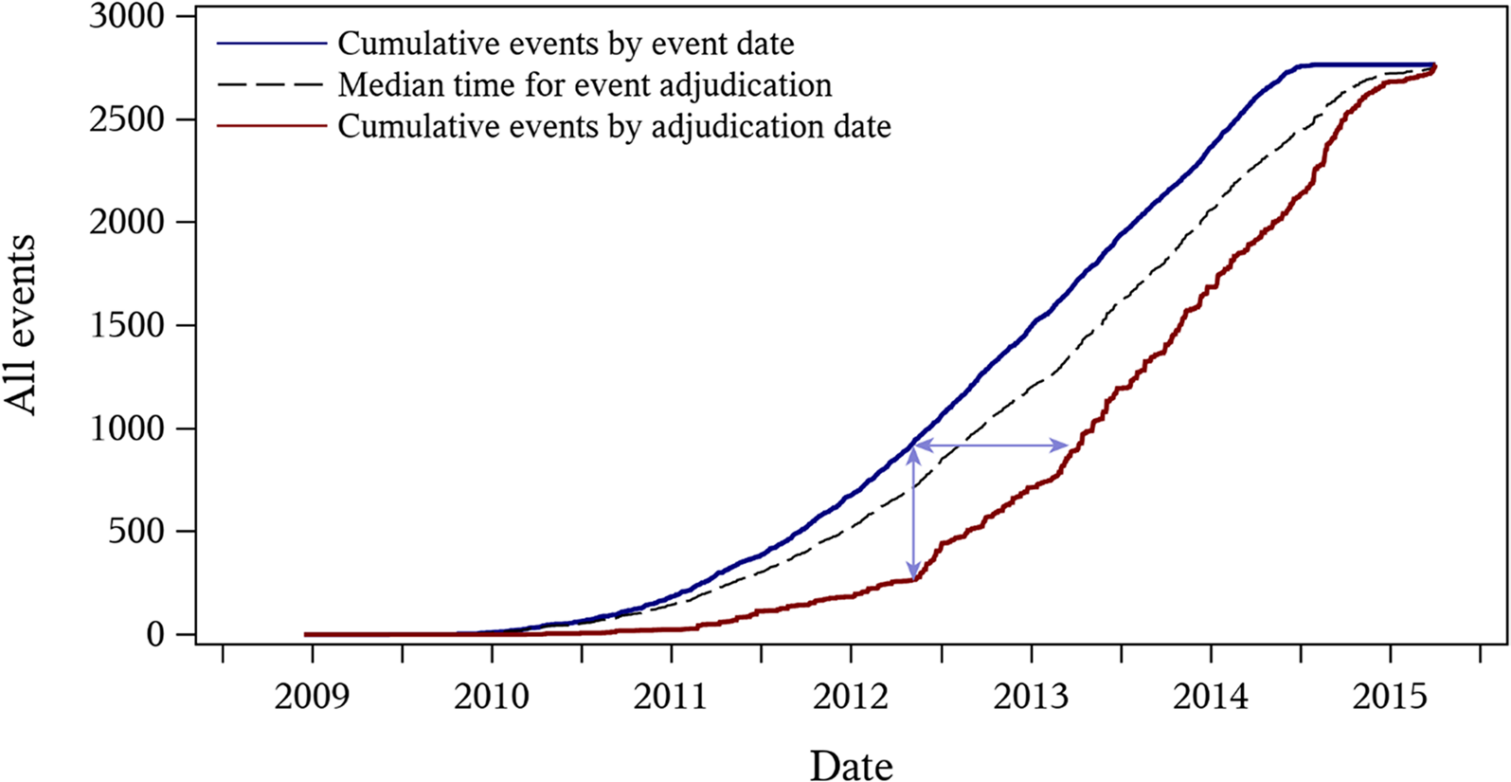
Measuring the adjudication gap. The temporal gap between event occurrence and event accrual (event recorded after completion of adjudication) is shown. The blue line shows the cumulative number of events of all-cause death, stroke, myocardial infarction, hospitalization for heart failure, and hospitalization for unstable angina by event date. The red line shows cumulative event numbers by adjudication date. The dashed line shows the median time for event adjudication. The space between the red and blue lines is the “adjudication gap,” which can be interpreted as the amount of time between the occurrence of events in real time and the lag time taken to complete adjudication (horizontal arrow) or the difference in number of reported and adjudicated events at a given time point (vertical arrow). The area between the blue and dashed lines represents events requiring less than the median time for adjudication, and the area between the dashed and red lines represents those requiring longer than the median time

The total adjudication time is shown in Table 1. Overall median (25th, 75th percentiles) times shortened during the life of the trial—recruitment: 355 (156–586) days; follow-up: 182 (117–283) days; close-out 101 (71–136) days—and differed by event type, with the shortest for hHF [126 (72–244) days] and longest for hospitalization for unstable angina [289 (149–514) days]. Improvements (shortening) in adjudication time windows were seen for the period between event occurrence and site reporting, and between site reporting and sending of the adjudication package for examination. Figure 1 depicts the “adjudication gap” for confirmed (first) primary outcomes, showing the time lag between event adjudication dates and actual event dates. At its widest, the adjudication gap was approximately 1 year (March 2013), but more typically was closer to 6–9 months, in keeping with the 6-month within-trial visit intervals.

**Table 1:** Adjudication time windows

Event subset	Event to site report (N = 2727)	Site report to package sent (N = 2730)	Package sent to adjudication decision (N = 2763)	Total adjudication time (N = 2760)
Overall	68 (29–137)	48 (19–107)	13 (4–30)	214 (122–407)
Trial phase				
Recruitment	83 (35–194)	47 (14–117)	8 (3–22)	355 (156–586)
Follow-up	64 (28–116)	50 (21–104)	14 (5–34)	182 (117–283)
Close-out	22 (13–43)	30 (24–64)	13 (3–31)	101 (71–136)
Event type				
MACE-4	74 (34–143)	56 (24–119)	17 (7–35)	244 (142–445)
Fatal/nonfatal MI	80 (40–167)	55 (23–122)	15 (7–29)	271 (140–493)
Fatal/nonfatal stroke	73 (38–124)	46 (20–97)	44 (26–70)	223 (154–372)
Hospitalization for unstable angina	84 (41–181)	50 (16–108)	10 (4–24)	289 (149–514)
Cardiovascular death	61 (19–142)	58 (23–123)	14 (6–29)	220 (130–406)
All-cause mortality	53 (17–131)	57 (24–123)	13 (6–27)	213 (124–386)
Hospitalization for heart failure	67 (34–116)	24 (7–61)	1 (0–3)	126 (72–244)

MACE-4: 4-point major adverse cardiovascular event comprising nonfatal myocardial infarction, nonfatal stroke, cardiovascular death, or hospitalization for unstable angina; MI: myocardial infarction

Days within phases of event accrual and adjudication are shown as median (25th, 75th percentiles). Total adjudication time includes the time between when the event occurred and when a final adjudication decision was rendered

### True event rate analysis

The Weibull shape parameter estimate, which shows whether hazard rates for each event type were increasing (shape parameter > 1), decreasing (< 1), or constant (= 1) over time, is shown for each event type in Table 2. Although the estimates exceeded 1 for ACM and CV death, the deviations were not so great as to require use of a Weibull rather than a constant hazard rate exponential model to accurately depict the data. Models adjusted for age did not change the shape parameter estimates (data not shown).

**Table 2:** Estimates of the Weibull shape parameter in models by event type

Event	Estimate (95% CI) for Weibull shape
MACE-4	0.96 (0.92–1.01)
All-cause mortality	1.14 (1.08–1.21)
Cardiovascular death	1.08 (1.01–1.16)
Myocardial infarction	0.96 (0.89–1.03)
Stroke	0.93 (0.85–1.03)
Hospitalization for unstable angina	0.90 (0.79–1.01)
Hospitalization for heart failure	0.98 (0.90–1.08)

MACE-4: 4-point major adverse cardiovascular event comprising nonfatal myocardial infarction, nonfatal stroke, cardiovascular death, or hospitalization for unstable angina

**Fig. 2:**
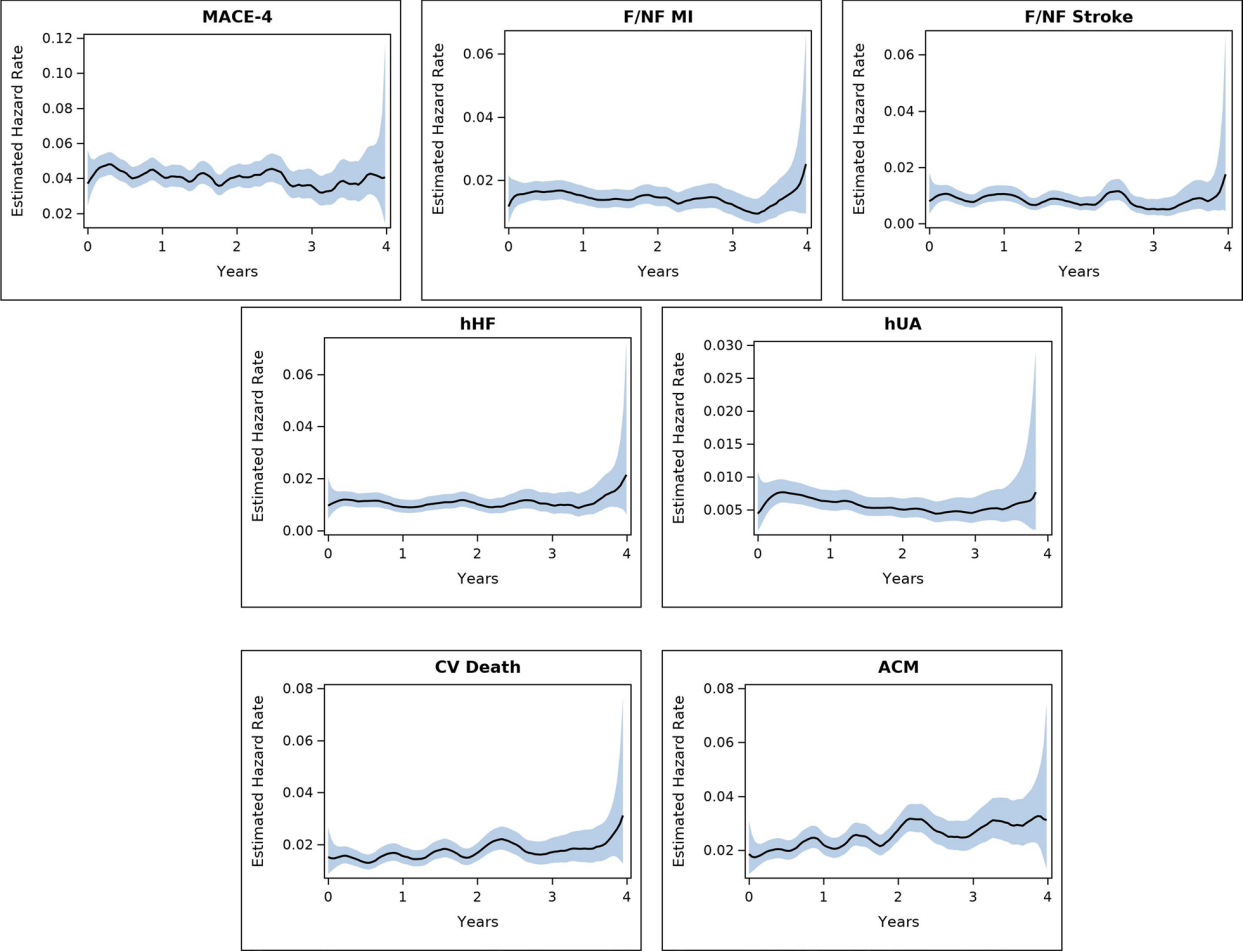
Real-time estimated hazard rates and 95% confidence intervals over the duration of the follow-up for 7 outcomes: MACE-4 [4-point major adverse cardiovascular (CV) event, comprising nonfatal myocardial infarction (MI), nonfatal stroke, CV death, or hospitalization for unstable angina (hUA)]; F/NF MI (fatal or nonfatal MI); F/NF stroke (fatal or nonfatal stroke); hHF (hospitalization for heart failure); hUA; CV death; and ACM (all-cause mortality)

Model fit was confirmed on review of results from all graphical plotting methods. Specifically, points in the plot of − log[S(t)] by time form a straight line through the origin, indicating a good fit for exponential model (Additional file 1: Fig. S1). Likewise, Weibull model plots show most points fall along the diagonal and within the confidence bands (Additional file 1: Fig. S2), and Epanechnikov hazard rate plots allow placement of a horizontal line that generally falls within the blue confidence bands (Fig. 2).

## Discussion

The TECOS data show that, for patients with T2D and known CV disease, the real-time hazard rates for MACE-4 and other important CV outcomes remained constant over the duration of the trial. Hazard rates for death (ACM and CV mortality) increased slightly over time, but not to an extent that indicates the need for complex modelling assumptions to predict events—exponential models that assume a constant hazard rate over time fit the data well. Examination of the within-trial event accrual pattern shows an “adjudication gap” that reflects the efficiency of trial processes for detecting events and confirming them with independent adjudication. Efficiency improved as the trial progressed, reflected by shorter adjudication times and impacted by event type.

Broadly stated, demonstration of a constant event hazard rate shows that trial participants were at an equal risk of experiencing a nonfatal CV event, including the MACE-4 composite, at all time points within the follow-up. This observation provides confidence in traditional modelling approaches for within-trial event rates. Even the event types with Weibull shape parameter estimates > 1 (CV death and ACM) were not sufficiently divergent from a constant hazard rate to require alternate assumptions. The reason for a small increase in hazard rate for fatal events is unclear and was not explained by adjusting the models for age. It is possible that a history of previous CV events, as required by the TECOS entry criteria, increases risk for subsequent death to a greater extent than for subsequent nonfatal events [3, 4].

With important trial events occurring at a constant rate, the next most pressing issue is to ensure robust trial processes to define, ascertain, and confirm relevant events. CVOTs benefit from internationally agreed definitions for most important CV events [5], and these definitions drive the elements of data collection. We propose here monitoring the adjudication gap during trials by quantifying the times between event incidence (actual event date), through event ascertainment (date the event is reported to the trial) and event confirmation (date the adjudication is completed). As shown, event hazard rate (and therefore, incidence) is constant and cannot be controlled. Addressing modifiable factors that affect the adjudication gap could improve event accrual efficiency. In TECOS, the adjudication gap was typically about 6 months in duration. We hypothesize that this was driven primarily by the frequency of trial visits at months 4 and 8 and semi-annually thereafter—a feature of the trial design that, if changed, might impact the adjudication gap. We observed an approximately 25% shortening of the time between overall event occurrence and site reporting from the trial recruitment phase [median time 83 (25th, 75th percentiles 35–194) days] to the closeout phase [22 (13–43) days]. Although not directly measured, it is possible that site familiarity with event reporting and associated documentation, the change in focus from recruitment to retention and then closeout, and continued training on the importance of “real-time” data entry all played a role in reducing this metric. Shortening of total adjudication time can also occur as adjudication committee operation processes become more efficient. While event rates remain constant, the absolute number of trial events rises over time with continued recruitment. As a consequence, the adjudication committee meets more frequently, quality control processes and adjudication training facilitate consistent review of events, and the turnaround times for the entire adjudication process shorten.

Critical decisions about trial design and resource allocation are affected by the adjudication gap. Understanding what influences the adjudication gap is an important part of clinical trial management and operations, and could have implications for key trial milestones. In an ongoing trial, the length of the adjudication gap affects how quickly a determination can be made that projected event rates are higher or lower than anticipated, which might prompt protocol amendments to the sample size trial entry criteria. In an event-driven trial, reliable forecasting of accrual time for the final events is required to effectively plan and conduct resource-intensive end-of-trial activities, such as monitoring visits and data cleaning. Monitoring of patient safety could also be hampered if underreporting, delayed reporting, or delayed adjudication of potential events impede the ability of a data and safety monitoring committee to review unblinded event data.

Interpretation of this analysis is limited by its inclusion of only TECOS data, making generalizability beyond populations defined by T2D and established CV disease unwise. Primary outcome event rates recorded in the placebo group of other completed T2D CVOTs vary widely, ranging from 24.2 per 1000 patient years in the Dapagliflozin Effect on Cardiovascular Events–Thrombolysis in Myocardial Infarction 58 trial to 58.7 per 1000 patient years in the Harmony Outcomes study [6, 7]. Understanding the impact of trial population on hazard rate over time requires additional study. Patterns of hazard rate over time may also differ in populations with lower CV risk, without established CV disease, with recent acute CV events, or with other comorbid conditions (e.g., chronic kidney disease). In addition, the median trial follow-up in TECOS was 3.0 years, limiting conclusions about longer-term event rate projections. Finally, factors influencing the adjudication gap may be unique to TECOS, its enrolled sites, and its trial conduct. However, we believe that the confirmation of constant hazard rate for important CV outcomes in patients with established CV disease and T2D is reassuring for clinical trial planning in this population. Similar analyses of event hazard rate over time should be conducted as more diverse populations, including those with mixed CV risk or characterized primarily by heart failure, are studied in CVOTs in diabetes.

## Supplementary Information

**Additional file 1. Fig. S1.** Plots of the negative log of KM survival distribution estimate (− log[S(t)]) versus time by event type. **Fig. S2.** Weibull probability plots with 95% confidence intervals by event type.

## Data Availability

Requests to access the data for this study from qualified researchers trained in human subject confidentiality protocols may be submitted at dcri.org/data-sharing.

## Abbreviations

ACM: All-cause mortality
CV: Cardiovascular
hHF: Hospitalization for heart failure
MACE-4: Major adverse CV event composite
TECOS: Trial Evaluating Cardiovascular Outcomes with Sitagliptin
T2D: Type 2 diabetes
