# Supplementary material for: Time-dependent event accumulation in a cardiovascular outcome trial of patients with type 2 diabetes and established atherosclerotic cardiovascular disease
Source: Cardiovasc Diabetol. 2023 Mar 28;22:72. doi: 10.1186/s12933-023-01802-x (PMC10054031; doi:10.1186/s12933-023-01802-x)

**SUPPLEMENTAL MATERIAL**

**Figure S1.** Plots of the negative log of KM survival distribution estimate (–log[S(t)]) versus time by event type

**Figure S2**. Weibull probability plots with 95% confidence intervals by event type

**Figure S1.** Plots of the negative log of KM survival distribution estimate (–log[S(t)]) versus time by event type


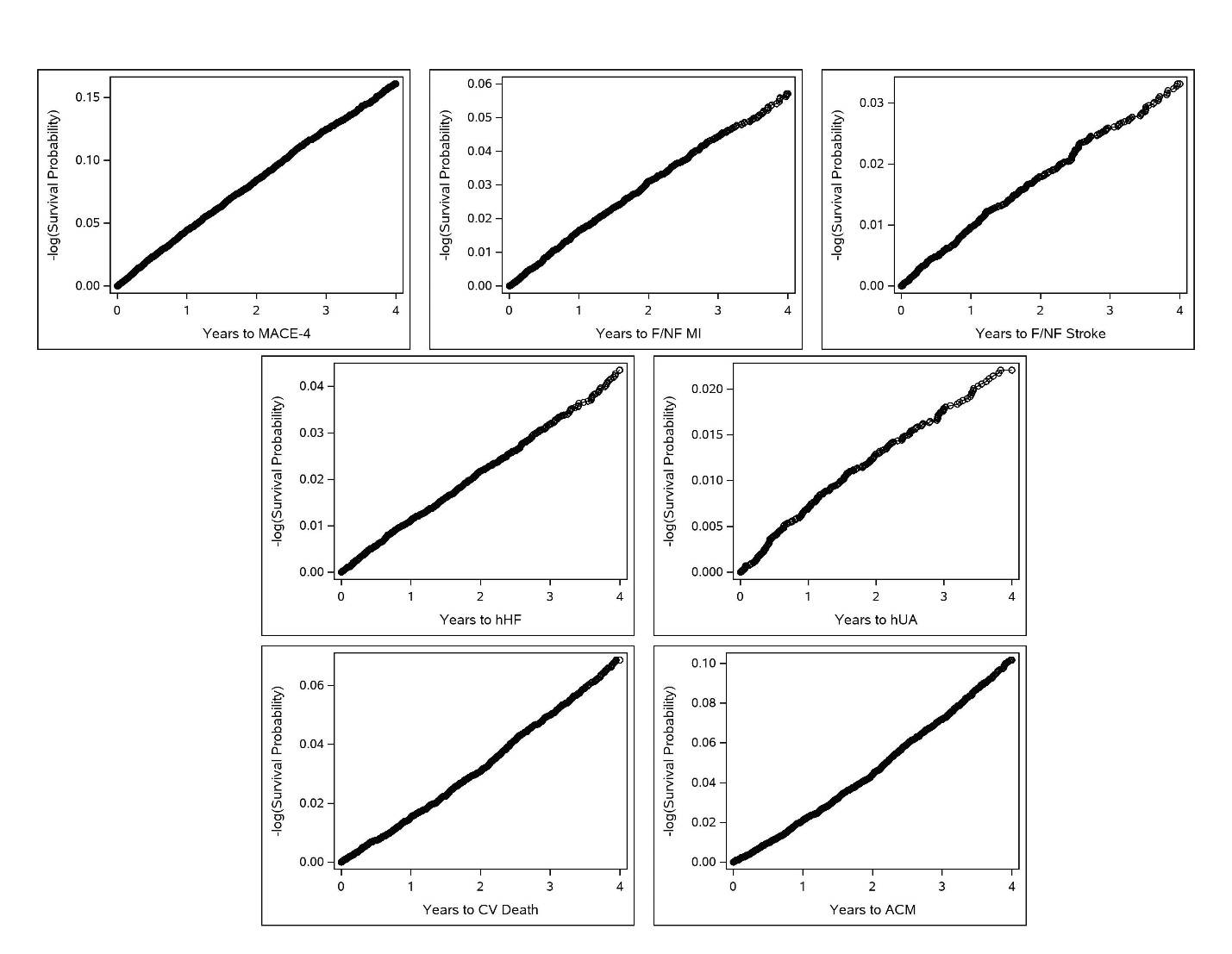


**Figure S2**. Weibull probability plots with 95% confidence intervals by event type


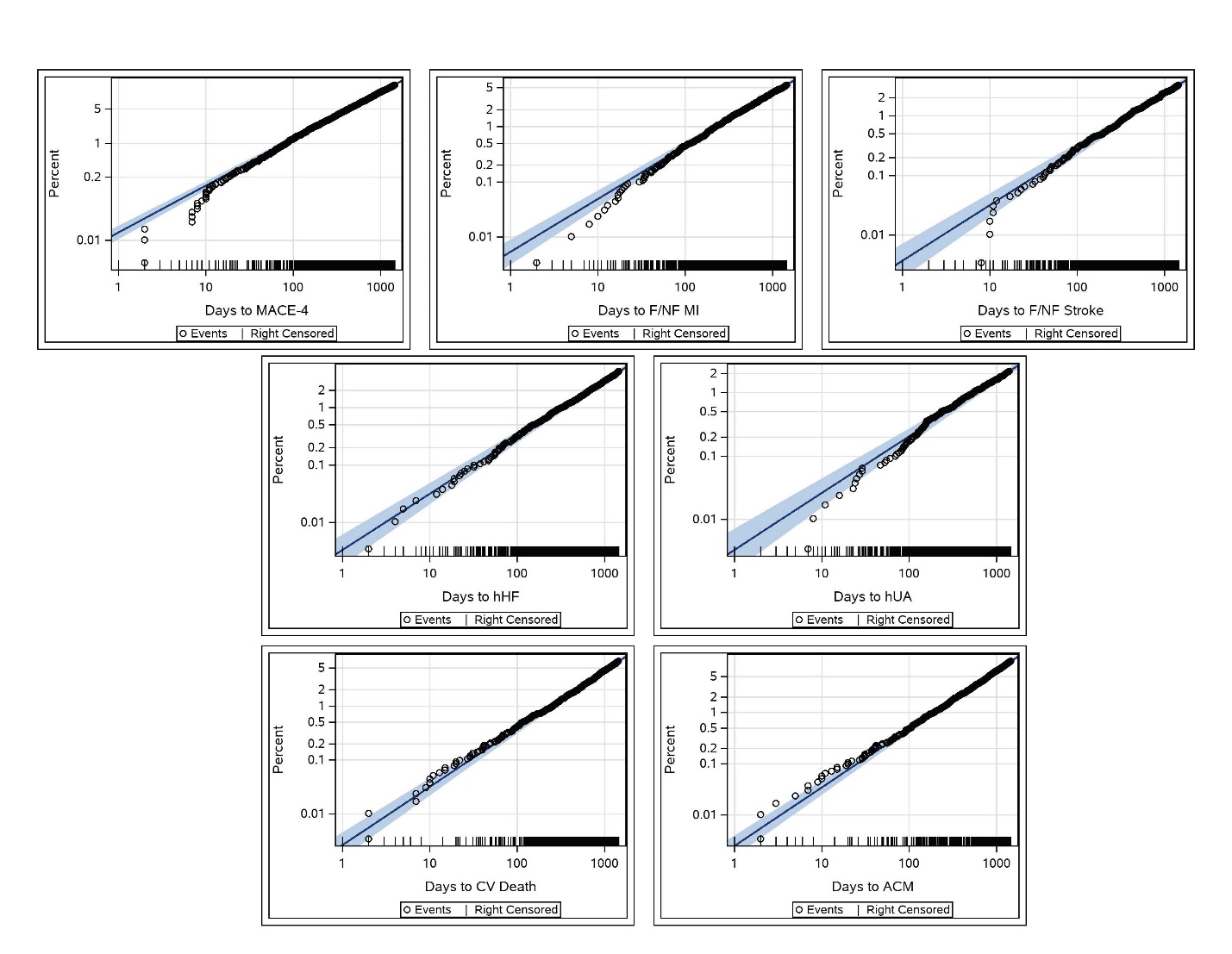

Supplement: Supplementary file 1 — Additional file 1. Fig. S1. Plots of the negative log of KM survival distribution estimate (− log[S(t)]) versus time by event type. Fig. S2. Weibull probability plots with 95% confidence intervals by event type. [file 12933_2023_1802_MOESM1_ESM.docx]
